# Characterising Skin Electrical Impedance Using Tape Stripping Methods: A Bioelectrical Study of a Porcine Model

**DOI:** 10.7759/cureus.66566

**Published:** 2024-08-10

**Authors:** Dylan Rowe, Mariam Rowe

**Affiliations:** 1 Medicine, School of Medicine, Griffith University, Brisbane, AUS; 2 Radiation Oncology, Faculty of Medicine, University of Queensland, Brisbane, AUS

**Keywords:** medical device, stratum corneum, porcine, electrical impedance, microneedle, skin

## Abstract

Background

Recent advancements in ultra-low power electronics and wireless devices have facilitated the widespread adoption of wearable technology for fitness and health monitoring, paving the way for personalized medicine. Microneedle-based devices, comprising small epidermal patches that penetrate the skin’s stratum corneum to potentially access biomarkers in the extracellular fluid of the viable epidermis, represent a promising innovation in this field.

Objectives

This project aimed to develop and validate a novel method to evaluate microneedle engagement in the skin in real-time. To our knowledge, there are no studies published to date that have characterized the electrical impedance of stratum corneum and epidermis using the tape stripping method to selectively remove cell layers. Additionally, no studies have been published comparing the electrical impedance of fresh to frozen-thawed porcine skin. The objective of this study was to develop and validate a novel method to evaluate microneedle engagement in skin, in real-time, that does not require processing of the tissue.

Methods

A tape stripping technique was employed to selectively remove the stratum corneum from fresh and frozen-thawed porcine skin samples which were then electrically characterized using an excitation frequency of 5 kHz with a peak Voltage of 1 V.

Results

This study demonstrated a mean impedance reduction of 97.08 ± 1.3 % for fresh porcine skin, and 98.04 ± 0.3 % for frozen-thawed porcine skin when transitioning from the surface stratum corneum to the viable epidermis. The correlation between the reduction of impedance and the number of tape strips across all 18 test sites was significant (r = 0.98, p < 0.00001). However, comparing the skin impedance of the fresh and frozen-thawed specimens showed poor equivalence, with the frozen-thawed sites approximately 5.5 times the impedance of the fresh sites before any tape stripping, and 4.19 times greater after 30 tape strips.

Conclusions

These findings suggest that monitoring for an interelectrode impedance decrease of greater than 95% between two projections of a microneedle device could provide a rapid and effective evaluation of skin engagement, crucial for advancing the development and clinical application of microneedle-based technologies in personalized medicine. The study also underscores the impact of the freeze-thaw process on the mechanical and electrical properties of skin, which is crucial for standardizing testing protocols.

## Introduction

Wearable technology is rapidly expanding in both research and industry. Popular devices such as smartwatches and fitness trackers use sensors on the skin's surface for electrocardiogram (ECG) and heart rate monitoring [1]. These sensors measure the changing potentials on the skin's outer surface, the stratum corneum.

Beneath the stratum corneum, the viable epidermis (10 to 40 micrometers deep) provides access to numerous biomarkers for potential medical diagnostics [2]. Microneedle-based devices offer a promising solution to bypass the stratum corneum. These devices utilize micro-projections to penetrate the skin’s outer barrier without reaching the nerve endings in the dermis, ensuring a relatively painless experience [3]. Accurate measurement of biomarkers within the viable epidermis is critical, but the thin layers make it challenging to evaluate penetration depth effectively.

During microneedle device development, various microstructure geometries, patch densities, application forces, and velocities are tested. These tests often use porcine skin samples to analyze penetration depth, retention force, uniformity, and ease of insertion. Targeting the viable epidermis involves penetration depths from 20 to 150 micrometres below the skin surface [2]. Common evaluation methods include optical coherence tomography [4], histology, fluorescence microscopy [5], scanning electron microscopy [6], confocal microscopy [7], and electrical impedance spectroscopy [8]. Among these, only electrical impedance spectroscopy offers real-time measurements and does not require further processing of tissue samples.

Skin, like other bodily tissues, is a non-homogeneous electrical conductor. The electrical characteristics of skin layers depend on tissue composition and moisture content. The stratum corneum, with 20% water content, is hydrophobic and highly resistive, while the viable epidermis, with 40% water, is hydrophilic and more conductive [9]. The viable epidermis is approximately 10 times more conductive than the stratum corneum [9]. This conductivity difference can be used to determine an electrode's contact layer [10]. Resnika et al. used a bioelectrical measurement technique consisting of a gold-plated micro-projection device as the penetrating electrode and a silver chloride electrode on the skin surface as the second electrode [9].

This study aimed to develop a novel method using electrical impedance measurements across the stratum corneum and viable epidermis to evaluate microneedle engagement into skin that does not require processing of the tissue. We used porcine ear skin as it is one of the most common human skin equivalents due to its similar cellular structure and mechanical characteristics [11,12].

## Materials and methods

Study design

Animal experiments were conducted in our institution’s testing laboratory in accordance with the regulations of the Australian Animal Ethics Committee (AEC). The need for ethical approval from the committee was not required as samples were sourced from an established food chain. Six fresh porcine ears were sourced from a local abattoir for this study. Three ears were tested while still fresh within five hours of animal slaughter; the remaining three ears were frozen within five hours of collection and stored at -20 °C until required. Prior to testing, the frozen ears were removed from storage and thawed at room temperature. All samples were cleaned with 70% isopropyl alcohol to remove visible contaminants and dried with a paper towel, then all hair was removed using an electric shaver.

Tape stripping

The established tape-stripping method was used to selectively remove layers of cells from the stratum corneum [13,14]. Three 2 cm² test sites, visually similar in colour and texture, were selected per ear specimen and marked with a permanent marker to ensure accurate and repeatable tape application. A fresh section of tape was cut from a roll of clear cellophane tape for each application. The tape was applied to the test site and held in place at 5 N for three seconds before being removed in one swift motion. Each subsequent piece of tape was applied after rotating 90 degrees. We used a total of 30 tape strips to remove the entire stratum corneum, as described by Konstantin et al. [15]. Stripping effectiveness was also assessed by visually inspecting the site for a shiny, wet appearance, as described by Lindemann et al. [16].

Electrical impedance measurement

**Figure 1:**
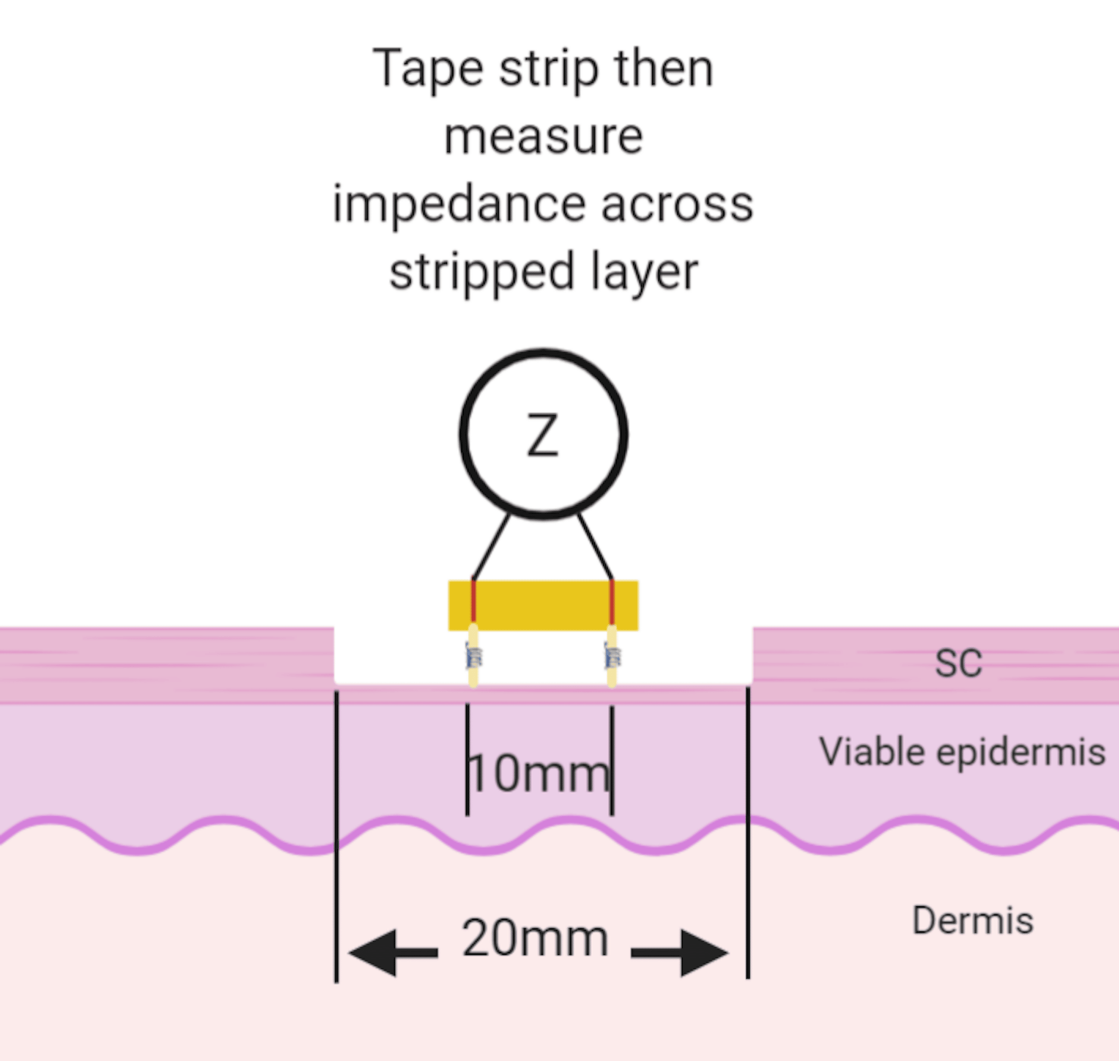
Tape stripping experiment on porcine skin, measuring the impedance of each stratum corneum layer. Custom electrode jig holding the two electrodes 10 mm apart. Z: Impedance measurement, SC: Stratum corneum Image created by the authors.

An AD5940 development board (Analog Devices Inc., Wilmington, MA, USA) with an ADICUP3029 microcontroller (Analog Devices Inc.) was programmed to measure complex impedance using a two-probe setup (Figure 1). The system was initialized to output a sine-wave signal at a frequency of 5 kHz with a peak amplitude of 1 V to reduce potential artefacts of the skin-electrode interface as described by Lu et al. [17]. The output signal was validated using an EDUX1052G oscilloscope (Keysight Technologies, Santa Rosa, CA, USA) before each experiment.

A custom jig was created to hold two electrodes 10 mm apart to ensure consistent spacing across experiments. The microcontroller was connected to the electrodes using alligator clips with leads 50 mm in length. The electrodes were applied to the porcine sample with a force of 5 N until each measurement was complete. The mean of three measurements was taken per recording. The serial data was captured using the 'RealTerm' serial terminal and processed with MATLAB (MathWorks, Natick, MA, USA).

Statistical analysis

We investigated the primary outcome of the correlation between impedance measurements and the number of tape strips. The statistical significance of impedance changes between successive tape strips was evaluated using paired t-tests. Pearson correlation coefficients were computed to assess the relationship between impedance reductions and the number of tape strips applied. As a secondary outcome, the skin electrical impedance of fresh and frozen-thawed porcine skin was compared to determine equivalence. All paired t-tests used a statistical significance set at p < 0.05. All data are presented as the mean ± standard deviation. Statistical analyses were performed using MATLAB.

## Results

**Figure 2:**
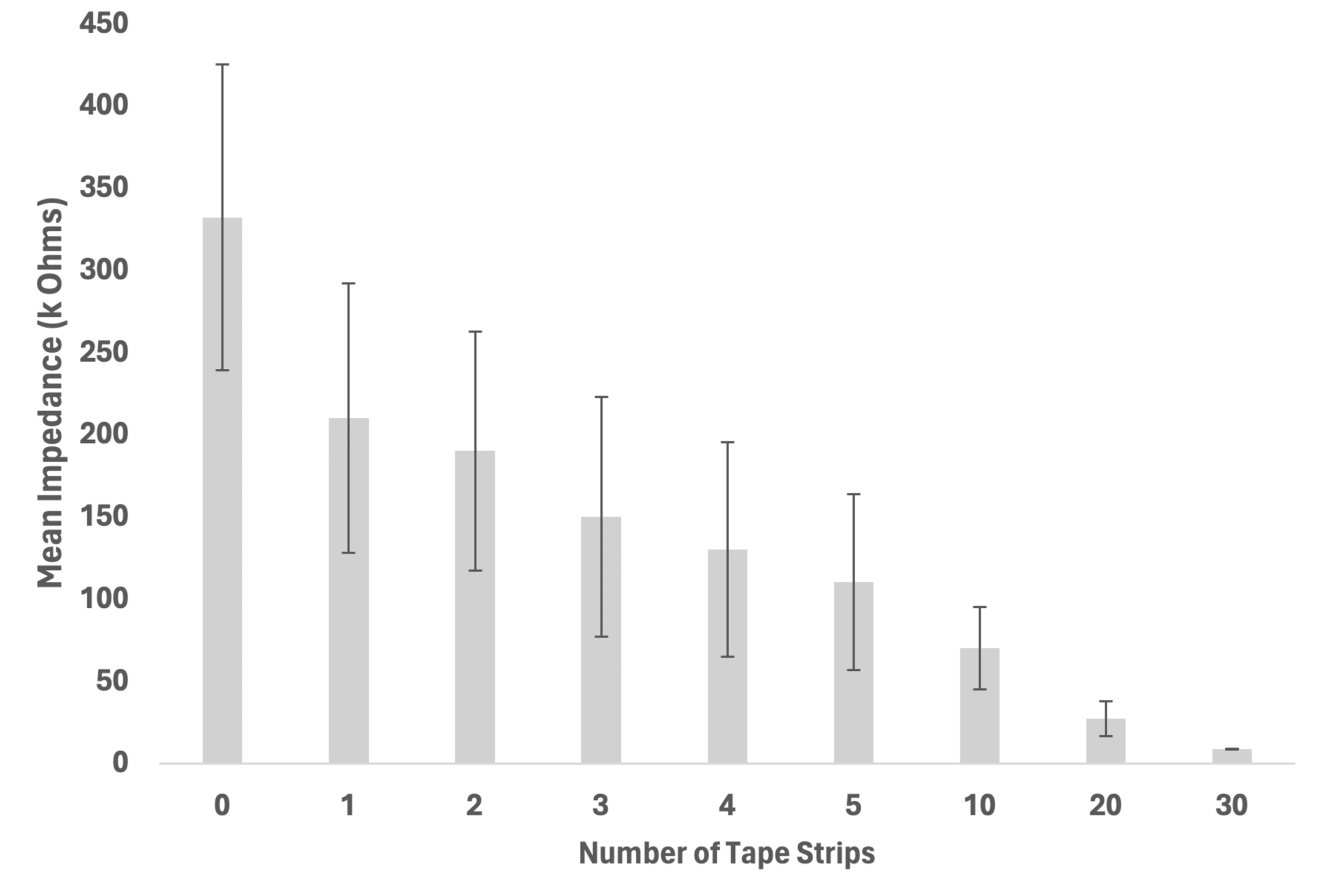
Stratum corneum electrical impedance with number of tape stripping on fresh porcine skin (n = 9, mean ± standard deviation)

For the nine fresh porcine test sites, the mean impedance measured prior to tape stripping was 332 ± 93 kΩ (Table 1). Figure 2 features a bar graph showing the impedance with standard deviation across all tape strips on the fresh porcine. The impedance measured after 30 tape strips was 8.57 ± 0.43 kΩ. The percentage change of impedance from 0 to 30 tape strips was 97.08 ± 1.3%. The reduction of impedance across all nine porcine sample sites per tape strip was significant (r = 0.98, p < 0.0001).

**Table 1:** The mean skin impedance before and after tape stripping and the total percentage of reduced impedance in fresh porcine skin. Data are presented as mean ± standard deviation

Variables (n = 18)	0 Tape strips	30 Tape strips	% Change	p-value
Impedance (kΩ)	332 ± 93	8.57 ± 0.43	97.08 ± 1.3	<0.0001

**Figure 3:**
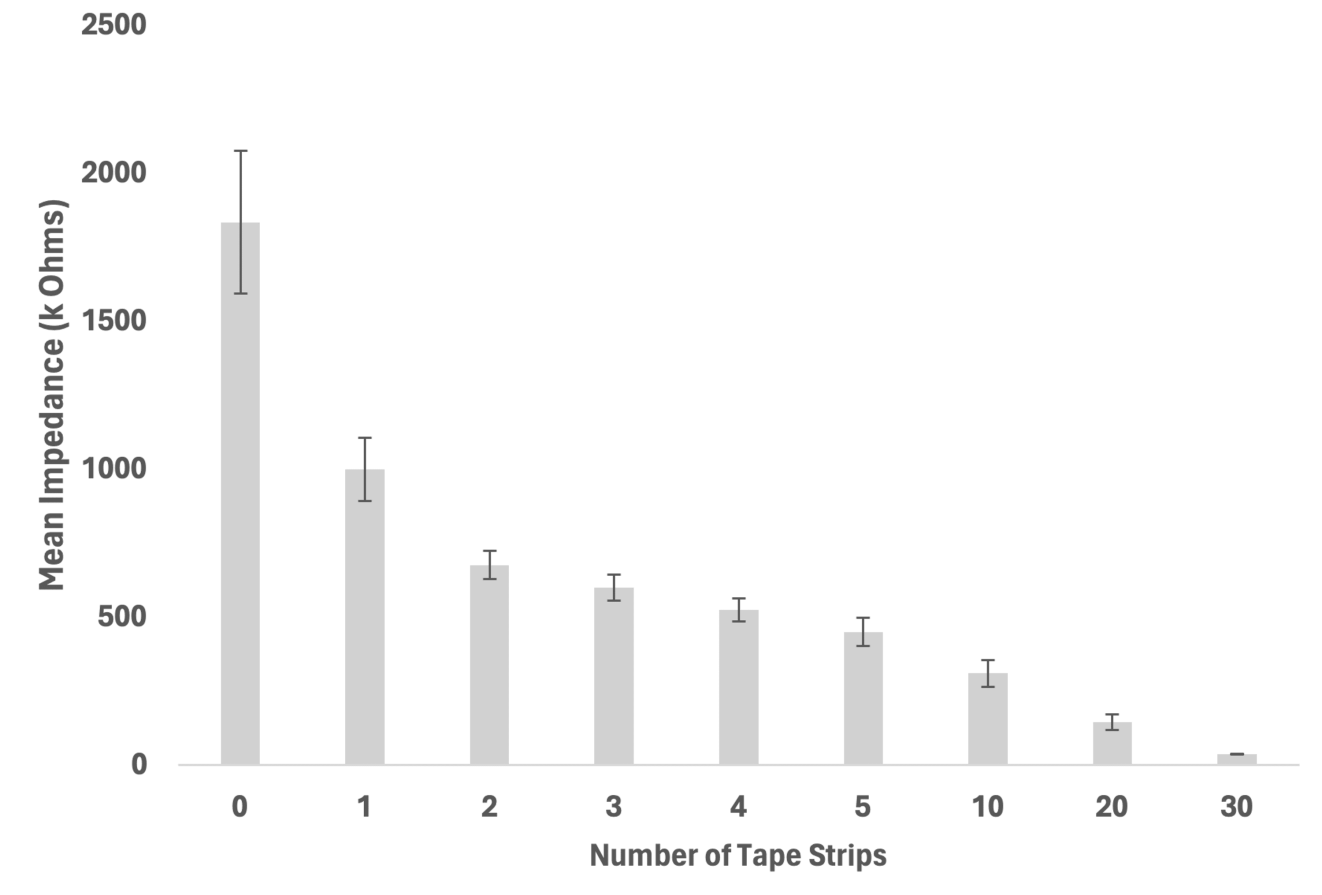
Stratum corneum electrical impedance with number of tape stripping on frozen-thawed porcine skin (n = 9, mean ± standard deviation)

For the nine frozen-thawed porcine test sites, the mean impedance measured prior to tape stripping was 1834 ± 240 kΩ (Table 2). A bar graph showing the impedance with standard deviation across all tape strips on the frozen-thawed porcine is shown in Figure 3. The impedance measured after 30 tape strips was 35.89 ± 1.62 kΩ. The percentage change of impedance from 0 to 30 tape strips was 98.04 ± 0.3%. The reduction of impedance across all nine frozen-thawed porcine sample sites per tape strip was significant (r = 0.98, p < 0.0001).

**Table 2:** The mean skin impedance before and after tape stripping and the total percentage of reduced impedance in fresh porcine skin. Data are presented as mean ± standard deviation

Variables (n=9)	0 Tape strips	30 Tape strips	% Change	p-value
Impedance (kΩ)	1834 ± 240	35.89 ± 1.62	98.04 ± 0.3	<0.0001

However, comparing the skin impedance of the fresh and frozen-thawed specimens showed poor equivalence, with the frozen-thawed sites approximately 5.5 times the impedance of the fresh sites before any tape stripping, and 4.19 times higher after 30 tape strips.

## Discussion

During the design and development of microneedle devices, various microstructure geometries, densities, application forces, and velocities are experimentally tested. Current methods of validating effective skin penetration require post-processing of the skin sample, such as when using histology. Postprocessing is time-consuming, inhibits rapid prototyping, and is not transferable to an end-consumer device. There are currently no known methods to evaluate uniform stratum corneum penetration in real-time during device application.

Our study used a tape stripping technique to selectively remove the stratum corneum from fresh and frozen-thawed porcine skin samples, which were then electrically characterized using an excitation frequency of 5 kHz with a peak voltage of 1 V. The impedance reduction curves of the frozen-thawed porcine samples showed a strong correlation across all nine sites (r = 0.98, p < 0.0001). Likewise, the impedance reduction curves of the fresh porcine samples showed a strong correlation across all nine sites (r = 0.98, p < 0.0001). However, when comparing the two samples, the impedance of the frozen-thawed sites was of greater magnitude than that of fresh porcine tissue. It is well established that some mechanical changes exist when freezing and thawing porcine skin samples, such as a lower Young’s modulus [11]. The differences in electrical properties between the two are not as well studied, but the results from this test suggest that the freezing and thawing processes may alter the electrical characteristics of the stratum corneum by increasing the impedance of both the stratum corneum and viable epidermis.

This study found that the impedance of the viable epidermis is less than 95% of the surface stratum corneum impedance when measured between two electrodes across each layer. It also suggests that by monitoring the electrical impedance between two isolated projections of a microneedle device, a real-time evaluation of microneedle engagement could be achieved. Extending this method to individually monitor all projections across a microneedle device has the potential to determine overall penetration uniformity. As this method does not require any post-processing of the tissue, such as histology, it can be refined during lab-based experiments and then incorporated into a final commercial device. For instance, a user could be instructed to press the microneedle device onto their skin, and once the impedance falls more than 95% from a surface measurement, the device could indicate a 'successful application' message.

A limitation known during this study pertains to the data acquisition device and excitation frequency used. Excitation frequencies applied at the surface electrodes vary across the literature depending on which characteristic of the skin is being measured [18]. It is also known that capacitive artefacts due to the electrode-skin interface can be introduced during measurements [19]. It is unknown whether these artefacts will differ when measured with a microneedle device compared to the electrode configuration used for this study. It is recommended that the study be reproduced using the final microneedle device intended for commercial development on human skin.

## Conclusions

This study presents a novel method for real-time evaluation of microneedle engagement in the skin using electrical impedance measurements. By employing a tape stripping technique to selectively remove the stratum corneum and analyzing impedance changes throughout cellular removal, we demonstrated a significant reduction in impedance (over 95%) when transitioning from the stratum corneum to the viable epidermis. The consistent impedance reduction observed in both fresh and frozen-thawed porcine skin samples highlights the potential of electrical impedance as a reliable and rapid indicator of microneedle penetration depth.

Our findings suggest that continuously monitoring impedance during skin penetration can provide immediate feedback on microneedle device performance, facilitating rapid prototyping and enhancing the development of microneedle-based technologies for personalized medicine. Moreover, our study underscores the impact of the freeze-thaw process on the mechanical and electrical properties of skin, which is crucial for standardizing testing protocols. Future research should focus on refining this impedance measurement technique and validating it with human skin samples to ensure broader applicability in clinical settings.
